# Depression Mediates the Relationship between Food Insecurity and Pain Interference in College Students

**DOI:** 10.3390/ijerph18010078

**Published:** 2020-12-24

**Authors:** Masataka Umeda, Sarah L. Ullevig, Eunhee Chung, Youngdeok Kim, Tanya J. Escobedo, Christopher J. Zeitz

**Affiliations:** 1 Department of Kinesiology, University of Texas at San Antonio, San Antonio, TX 78249, USA; eunhee.chung@utsa.edu; 2 Coordinated Program of Dietetics, University of Texas at San Antonio, San Antonio, TX 78249, USA; sarah.ullevig@utsa.edu; 3 Department of Kinesiology and Health Sciences, Virginia Commonwealth University, Richmond, VA 23284, USA; kimy13@vcu.edu; 4 Department of Psychology, University of Texas at San Antonio, San Antonio, TX 78249, USA; tanya.escobedo@my.utsa.edu (T.J.E.); christopher.zeitz@my.utsa.edu (C.J.Z.)

**Keywords:** food security, depression, pain, college students, stress

## Abstract

Food insecurity (FI) typically produces unfavorable health conditions. Research shows the high prevalence of FI among college students, and depression is one of the adverse effects of FIamong them. It is possible that FI may increase the risk of pain via depression; however, it is currently unclear whether FI is linked to pain among college students. Therefore, this study compared pain experiences between students with and without FI, and examined the relationship between FI, depression, and pain. One hundred seventy-six college students at a Hispanic-serving institution in the southwestern region of US completed self-report measures to assess FI, depression, pain severity, and pain interference. Results indicated that approximately 24% of the students were categorized as food insecure, and those students scored higher on pain interference compared to food-secure students. FI was positively associated with depression and pain interference scores, and depression scores were positively associated with pain interference scores. The mediation analyses based on the counterfactual framework demonstrated a significant mediation effect of depression, where 50.59% of the total effect of FI on pain interference was attributable to the depression. These results suggest that FI extends its negative effects into pain interference among college students, but better management of depression may help alleviate the effects of FI on pain interference.

## 1. Introduction

Food insecurity (FI) is characterized by limited access to safe and healthy food for active, healthy living due to a lack of money and other resources [1]. Data show that the prevalence of FI has been decreasing gradually in the last few years, but the recent data still indicate the existence of a large number of American households who experienced FI during the year of 2017 (approximately 12%, 15 million households) [1]. It has been reported that those households with incomes near or below the poverty line and Black- and Hispanic-headed households are at greater risk of FI [1], suggesting that limited financial ability and racial/ethnic backgrounds could be potential factors that are linked to FI. These observations lead us to speculate that continuous escalation of college tuition and fees over the decades in US [2] may place college students at increased risk of FI. A systematic review summarizing the prevalence of FI in postsecondary campuses supports the speculation by showing the high prevalence of FI among students attending US colleges (average = 32.9%, range = 14.1 to 58.8%) [3].

Not surprisingly, it is documented that FI is generally linked to poor health conditions, such as hypertension, diabetes, depression, etc. [4,5], and the adverse effects of FI on health and healthful behaviors are observed among college students as well, such that food-insecure college students are more likely to rate their health unfavorably, experience stress and depression, and engage in unhealthy dietary habits and less physical activity (PA) behaviors compared to food-secure college students [3,6,7,8,9]. Given the high prevalence of FI among college students [3] and continued escalation of college tuitions and fees [2], these observations clearly indicate that college students are susceptible to poor health due to FI.

Previous studies demonstrate a link between FI and depression among college students [6,8], and it is possible that FI may be linked to other health outcomes via depression among them. For example, it is well known that depression is linked to chronic pain: cross-sectional evidence indicates that depression is more prevalent among individuals with chronic pain compared to pain-free individuals [10], whereas longitudinal evidence indicates that depression serve as risk factor of developing chronic pain in the future [11,12]. Together, the findings suggest that individuals with FI may be at greater risk of experiencing pain conditions via depression. However, it is currently unclear whether adverse health effects of FI extend into pain among college students. It is possible that pain can be detrimental for college students to function academically and socially, suggesting a need for more research to better understand the link between FI, depression, and pain among college students.

Therefore, the present study aimed to compare pain experiences between students with and without FI, and examine the relationship between FI, depression, and pain among college students. Furthermore, the present study aimed to test the potential mediating role of depression in the relationship between FI and pain. Based on the findings from past research, it is first hypothesized that those students with FI would experience more pain compared to those without FI. It is also hypothesized that FI would be positively associated with depression and pain, whereas depression would be positively associated with pain. Furthermore, depression would play a mediating role of the relationship between FI and pain.

## 2. Methods

### 2.1. Participants

Participants in this study were undergraduate students who were enrolled in an introductory kinesiology course in Fall 2017 at a state university in southwestern region of US. The university is designated as a Hispanic-serving institution, and enrolls socio-culturally diverse student populations. The institutional database indicated that 53% of students were Hispanic, which made up the largest racial group among the student population in the fall 2017 semester [13]. The Hispanic student population was followed by 24% of White, 9% of African American, and 5% of Asian, and these four racial groups made up more than 90% of the total student population in the semester (N = 30,768, 85% undergraduate students).

During the semester, 190 students out of 199 students who were enrolled in the course completed several questionnaires to evaluate their health as a class assignment, and the questionnaires were used as data for the present study. Among 190 students, 14 students had missing values in the primary variables of interest (FI, depression, and pain). Therefore, those cases were excluded from the analyses. To use the assignment as data for scientific investigation, we followed the Institutional Review Board (IRB)’s instructions regarding the ethical conduct of research, and then received an IRB approval for the investigation.

### 2.2. Procedures and Measures

The students completed the questionnaires described below, to evaluate their health and health-related behaviors, such as food security, depression, recent pain experiences, regular PA, as well as general demographic information. Trained research staff gave brief instructions on the questionnaires, and asked that the students answer the questions as honestly and accurately as possible.

*Food Security*: Food security was assessed using the USDA adult food security survey module [14]. The survey consists of 10 questions regarding the students’ financial ability to secure food during the last 30 days (e.g., “*I worried whether my food would run out before I got money to buy more*”). The students answered the questions based on (1) how often such statements were true for them, (2) whether they experienced food crisis (e.g., *cutting the size of meals or skip meals because there was not enough money for food*), and (3) how many days they experienced such food crisis. The survey was scored based on the USDA scoring protocol, such that higher scores, with a range of 0 and 10, indicate more severe food insecurity. The students were then categorized based on the scores into the following four groups: high food security, marginal food security, low food security, and very low food security. Consistent with the scoring protocol, the high food security and marginal food security groups were combined as food secure group, whereas the low food security and very low food security groups were combined as food insecure group.

*Depression*: Symptom severity of depression was evaluated using the Beck Depression Inventory II (BDI-II) [15]. The BDI-II is a validated self-report questionnaire to quantify depressive symptoms, and consists of 21 items that assess the psychological (e.g., hopelessness), cognitive (e.g., guilt), and physical (e.g., change in appetite) domains of depressive symptoms. Each item is rated on a 4-point scale ranging from 0 to 3, and the students marked one out of the four responses that best described their symptom. Scores range from 0 to 63 points, with higher scores indicating more severe depressive symptoms.

*Recent Pain Experiences*: The following two questions were adopted from the Bodily Pain Scale (BDS) from SF-36 Health Survey [16] to ask pain severity and pain interference during the last 4 weeks, respectively: “*How much bodily pain have you had during the past 4 weeks*?” and “*During the past 4 weeks, how much did pain interfere with your normal work*?” The former was rated with 6 responses (1: none, 2: very mild, 3: mild, 4: moderate, 5: severe, and 6: very severe), whereas the latter was rated with 5 responses (1: not at all, 2: a little bit, 3: moderately, 4: quite a bit, and 5: extremely). The two response formats show that greater numbers indicate greater pain severity and pain interference.

*Demographics and PA Behaviors*: Demographic- and health-related questions were asked in the demographic and health questionnaire. The students self-reported age, height (cm), weight (kg), gender, and race/ethnicity (American Indian/Alaskan Native, Asian, Black/African American, Hispanic/Latino, Native Hawaiian/Other Pacific Islander, White, and others). Due to a small number of American Indian/Alaskan Native, Asian, and Native Hawaiian/Other Pacific Islander individuals and individuals of other racial/ethnic backgrounds, these racial/ethnic minority groups were collapsed into one group, and categorized as others. This data treatment resulted in creating the following four racial/ethnic categories: Black/African American, Hispanic/Latino, White, and Others. Body mass index (BMI) was calculated using the following formula: BMI = weight (kg)/height (m)^2^. In addition, the students also self-reported if they were physically active enough to meet the following physical activity guidelines for adults [17]: engaging in moderate intensity aerobic PA for ≥150 min per week, vigorous intensity aerobic PA for ≥75 min per week, or an equivalent combination of moderate and vigorous intensity aerobic PA in addition to muscle-strengthening activities ≥2 days per week.

### 2.3. Statistical Analyses

Descriptive statistics were calculated for demographic and study variables across the food security groups. The mean differences of continuous variables (i.e., age, BMI, pain severity, pain interferences, and depression score) were examined using an independent samples t-test and the differences in the frequency distribution of categorical variables (i.e., gender, race/ethnicity, and PA) were examined using a chi-square test of independence.

**Figure 1:**
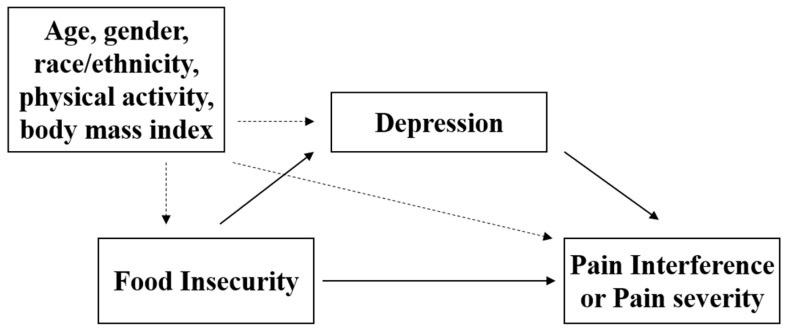
Hypothesized mediation model depicting the direct and indirect paths of food insecurity on pain throughout the depression after adjusting for study covariates.

In order to examine the potential mediating role of depression on the relationship between FI and pain, we constructed a linear regression model examining (1) FI as an independent dummy variable predicting pain as the dependent variable; (2) FI as an independent dummy variable predicting depression score as the dependent variable; and (3) depression as an independent variable predicting pain as the dependent variable. All models were adjusted for study covariates including age, gender, race/ethnicity, BMI, and PA. A follow-up mediation analysis was performed under the counterfactual framework [18] to estimate the amount of the total effect of FI on pain mediated by depression (see Figure 1, depicting a hypothetical mediation model). Specifically, the total effect was decomposed into the four components [19], including a controlled direct effect due to neither mediation nor interaction (i.e., the effect of FI on pain in the absence of the mediator, the depression), a reference interaction effect (i.e., the effect solely due to the interaction between FI and depression), a mediated interaction effect due to both mediation and interaction (e.g., the interaction of FI with depression that has been caused by FI), and a pure indirect effect (i.e., the effect of FI on pain purely mediated through depression). Additionally, the first and last two components were combined into the conventional 2-way decomposition, natural direct (i.e., controlled direct effect + reference interaction effect) and indirect effects (i.e., mediated interaction effect + pure indirect effect). The percentage of the effects accounted for by each component was calculated by dividing the estimate of each component by the total effect. A regression-based approach was used for parameter estimations of the counterfactual mediation analysis using the CAUSALMED procedure in SAS v9.4 (SAS Institute, Cary, NC). Bias corrected 95% confidence intervals were estimated via bootstrapping with 1000 replications. The analysis was adjusted for the study covariates (i.e., age, gender, race/ethnicity, BMI, and PA) based on the assumptions of no unmeasured confounders for the identification of the mediation effects [20]. The significance level was set at a familywise α = 0.05 for the analyses.

## 3. Results

Descriptive statistics of the final analytic sample (*n* = 176) are presented in Table 1. The results showed that the sample were generally young (*M_Age_* = 19.16 *SD* = 2.03), and included 24.43% (*n* = 43), who were found to be food-insecure at least some time during the last 30 days. Relatively large portions of final sample were male (54.55%), Hispanic/Latino (48.30%), and reported meeting current PA guidelines (54.55%). There were no statistically significant associations between FI and demographic characteristics of final sample (*P’s* > 0.05).

On average, the participants with FI (*M_Pain Interference_* = 1.81; *SD* = 0.94) had significantly higher pain interference scores (*p* = 0.006) when compared to the food-secure participants (*M_Pain Interference_* = 1.44; *SD* = 0.68). However, the differences in pain severity scores were not statistically significant (*p* = 0.251), whereas the differences in depression scores were only marginally significant (*p* = 0.064).

The results from a series of regression analyses are presented in Table 2. After controlling for study covariates, FI was a significant predictor of pain interference (*b* = 0.38; 95% CI = 0.11, 0.65) and depression (*b* = 2.47; 95% CI = 0.20, 4.74), but not of pain severity (*b* = 0.23; 95% CI = −0.17, 0.63). Similarly, depression was significantly associated with pain interference (*b* = 0.04; 95% CI = 0.02, 0.06) explaining 10.52% of variability of pain interference, but not with pain severity (*b* = 0.03; 95% CI = −0.00, 0.06).

Table 3 presents the results of mediation analyses based on the counterfactual framework. The 4-way decomposition of the effects of FI on pain interference showed that 55.69% of total effect was attributed to the controlled direct effect (*b* = 0.18; 95% CI = −0.01, 0.50), while 26.72% and 23.87% were attributable to mediated interaction (*b* = 0.09; 95% CI = −0.03, 0.32) and pure indirect effects (*b* = 0.08; 95% CI = 0.01, 0.21), respectively. When decomposing the total effect into the conventional 2-way components, 49.41% of the total effects were attributed to a natural direct effect (*b* = 0.16; 95% CI = −0.03, 0.32) and the remainder 50.59% were due to natural indirect effect (*b* = 0.16; 95% CI = 0.01, 0.42).

The total effect of FI on pain severity was not statistically significant (*b* = 0.15; 95% CI = −0.25, 0.67). After accounting for the interaction effects, 25.60% and 28.93% of total effects were attributable to controlled direct effect (*b* = 0.04; 95% CI = −0.39, 0.59) and pure indirect effect (*b* = 0.04; 95% CI = −0.02, 0.17), respectively. The natural direct and indirect effects accounted for 11.64% and 88.36% of the total effects; yet, both effects were not statistically significant.

## 4. Discussion

The present study estimated the prevalence of FI among college students who were attending a Hispanic-minority institution, and then compared pain scores between students with and without FI. Furthermore, the present study examined the relationship between FI, depression, and pain, and then tested the potential mediating role of depression in the link between FI and pain interference among college students. The results first showed that 24% of our study sample were categorized as FI, but racial/ethnic backgrounds of students were not linked to the FI prevalence. Those students with FI report experiencing greater pain interference compared to those students without FI. The results also showed that FI was positively associated with depression and pain interference, whereas depression was positively associated with pain interference. Follow-up mediation analyses showed a significant mediation effect of depression, where 50.59% of the total effect of FI on pain interference was mediated by the depression. Together, the present study adds to the current literature regarding FI and health among college students by showing that FI extends its negative health effects into pain interference, but better management of depression may alleviate the pain interference that food-insecure college students suffer from.

The present study was the first to report the adverse effects of FI on pain interference among college students. Investigating underlying mechanisms of the link between FI and pain interference is beyond the scope of this study; however, the literature suggests some potential explanations for the link between FI and pain interference. It has been shown that food-insecure college students were more likely to report experiencing stress and depression compared to food-secure college students [6,8], and medical research shows that such psychosocial stress hinders recovery from surgeries [21]. Additionally, it is well known that nutrition plays a vital role in recovery from injuries [22,23]. These observations collectively suggest that FI may potentially increase pain interference via slow recovery from events that cause pain, due to chronic exposure to psychosocial stress and/or lack of access to adequate nutrition. However, given that the present study is the first to report the findings, more research is needed in the future to replicate and examine the relationship between FI and pain interference among college students.

A previous study indicated that food-insecure college students were less likely to engage in healthful behaviors, such as PA, compared to food-secure college students [6]. The present study showed that food-insecure and food-secure college students were generally comparable in the PA behaviors; therefore, the findings were not in agreement with the previous study. The disagreement between the two studies is probably due to the fact that participants in the present study were those who were enrolled in a kinesiology course. Previous studies showed that the prevalence of self-reported sufficient aerobic PA to meet the similar PA guidelines in US young adults ranged from 52% to 67% [24,25]. The PA data in the present study indicated comparable prevalence of self-reported sufficient PA to meet the PA guidelines (food-insecure students: 60.47% and food-secure students: 52.63%). These observations indicate that most participants in the present study were sufficiently physically active, regardless of FI status, and such differences in study populations between the studies are likely to explain the disagreement.

The results indicated that approximately 24% of the students experienced FI. While the prevalence of FI is within a range of the reported prevalence of FI among students attending US colleges [3], the prevalence is still substantially higher than the reported prevalence of FI among American households [1]. It is currently unclear what may be responsible for the higher prevalence of FI among college students compared to general American households. However, it has been suggested that college students’ frequent use of money for nonfood items may place them at greater risk of FI [26,27]. Additionally, one study indicated that food-insecure college students showed a lack of confidence to cook, and meal preparation experiences compared to food-secure college students [28]. Together, these observations suggest that lack of general life skills (e.g., financial management, cooking/food preparations skills) may partially explain the higher prevalence of FI among college students. On the other hand, many universities and college campuses are establishing campus food pantries to address FI among college students [29,30]. Therefore, enhancing coordination of college and universities resources, improving access to campus mental health care services and food pantries, and providing more opportunities to learn such general life skills may help reduce the high prevalence of FI and FI-related health problems among college students.

There are several limitations of the current study. First, the sample size of the study was small, in comparison to the size of the total student population, and the study participants were limited to those who were enrolled in a kinesiology course. A larger sample of students of more diverse academic backgrounds, and PA levels will add methodological strength to the future research to better describe food-insecure college students. In fact, pain severity scores, pain interference scores, and depression scores were generally low in participants of this study, suggesting that effects of FI on these variables may have been minimized among physically active college students. It is currently unclear, however, whether FI may be more detrimental among physically inactive, food-insecure college students compared to physically active, food-insecure college students. Furthermore, PA was assessed subjectively using self-report questionnaires in the present study. Previous research shows the existence of large disparities in the prevalence of sufficient PA to meet the guidelines between subjective and objective assessments of PA [25]. Additionally, the present study did not incorporate any measure to quantify the participants’ eating habit, which is likely to impact PA behaviors and general health. Adding the measures of eating habit and PA behaviors, such as objective measures to evaluate PA, will likely allow future research to examine the FI and health more broadly. Lastly, the counterfactual framework-based mediation analysis has a strong assumption about ‘no unmeasured confounding’ for the exposure-outcome, mediator-outcome, and exposure–mediator relationships. Although we adjusted for study covariates in the analyses, there could be residual or unmeasured confounding from other factors.

## 5. Conclusions

The present study was the first to report that food-insecure college students experienced more pain interference compared to food-secure college students, and the link between FI and pain interference was mediated by depression. These findings suggest an importance of campus-wide efforts, such as campus pantry, to eradicate FI among college students, and depression management to minimize the effects of FI on pain interference.

## Figures and Tables

**Table 1:** Descriptive Statistics of Study Sample by Food Security Status.

	Total	Food Insecure	Food Secure	*p*-Value ^a^
*n* (%)	176 (-)	43 (24.43%)	133 (75.57%)	
Age (years)	19.16 (2.03)	19.33 (1.61)	19.11 (2.15)	0.538
Gender (*n*, %)				0.873
Male	96 (54.55%)	23 (53.49%)	73 (54.89%)	
Female	80 (45.45%)	20 (46.51%)	60 (45.11%)	
Race/Ethnicity (*n*, %)				0.398
African American	28 (15.91%)	10 (23.26%)	18 (13.53%)	
Hispanic/Latino	85 (48.30%)	21 (48.84%)	64 (48.12%)	
White	28 (15.91%)	5 (11.63%)	23 (17.29%)	
Other	35 (19.89%)	7 (16.28%)	28 (21.05%)	
Physical activity (*n*, %) ^b^				0.370
Sufficient	96 (54.55%)	26 (60.47%)	70 (52.63%)	
Insufficient	80 (45.45%)	17 (39.53%)	63 (47.37%)	
Body mass index (kg/m^2^)	24.53 (4.43)	24.25 (4.09)	24.62 (4.55)	0.633
Bodily pain scale ^c^				
Pain severity	2.49 (1.13)	2.67 (1.32)	2.44 (1.13)	0.251
Pain interference	1.53 (0.77)	1.81 (0.94)	1.44 (0.68)	0.006
Depression score ^d^	7.59 (6.34)	9.21 (5.58)	7.09 (6.49)	0.064

Note. Values are mean (standard deviation) for a continuous variable and n (%) for a categorical variable. ^a^ *p*-values from the between-group comparison using an independent sample t-test for a continuous variable and a chi-square test of independence for a categorical variable. ^b^ The categorization was based on self-reported physical activity (PA) levels meeting current PA, both aerobic and muscular strengthening, guidelines. ^c^ Greater scores on pain severity (ranges 1–6) and pain interference (ranges 1–5) indicate greater pain severity and pain interference. ^d^ Greater depression score (ranges 0–63) indicates severe depressive symptoms.

**Table 2:** The Relationships between Food Insecurity, Pain Measurements and Depression Score ^a^.

Models ^b^	Valid *n* ^c^	b (95% CI) ^d^	Semi-Partial ω^2^	*p*-Value
Food insecurity → Pain interference	173	0.38 (0.11, 0.65)	3.77%	0.026
Food insecurity → Pain severity	173	0.23 (−0.17, 0.63)	0.02%	0.264
Food insecurity → Depression	169	2.47 (0.20, 4.73)	2.06%	0.033
Depression → Pain interference	166	0.04 (0.02, 0.06)	10.52%	<0.001
Depression → Pain severity	166	0.03 (−0.00, 0.06)	1.34%	0.072

^a^ The linear regression model was established to examine each association after controlling for study covariates including age, gender, race/ethnicity, physical activity, and body mass index. ^b^ Food secure group was a referent category of food insecurity variable. ^c^ Missing data were listwise deleted from each model. ^d^ Unstandardized regression coefficient and 95% confidence interval.

**Table 3:** The Results of Mediation Analyses Based on the Counterfactual Framework (*n* = 166) ^a^.

	b (95% CI) ^b^	Proportion (%) ^c^
Outcome: Pain interference		
Total effect	0.32 (0.01, 0.70)	-
4-way decomposition		
Controlled direct effect	0.18 (−0.08, 0.50)	55.69%
Reference interaction	−0.02 (−0.13, 0.02)	−6.28%
Mediated interaction	0.09 (−0.03, 0.32)	26.72%
Pure indirect effect	0.08 (0.01, 0.21)	23.87%
2-way decomposition		
Natural direct effect ^d^	0.16 (−0.10, 0.48)	49.41%
Natural indirect effect ^e^	0.16 (0.01, 0.42)	50.59%
Outcome: Pain severity		
Total effect	0.15 (−0.25, 0.67)	-
4-way decomposition		
Controlled direct effect	0.04 (−0.39, 0.59)	25.60%
Reference interaction	−0.02 (−0.16, 0.02)	−13.96%
Mediated interaction	0.09 (−0.07, 0.37)	59.43%
Pure indirect effect	0.04 (−0.02, 0.17)	28.93%
2-way decomposition		
Natural direct effect ^d^	0.02 (−0.42, 0.60)	11.64%
Natural indirect effect ^e^	0.13 (−0.03, 0.41)	88.36%

^a^ The estimates were adjusted for study covariates including age, gender, race/ethnicity, physical activity, and body mass index. ^b^ Bootstrapped bias corrected 95% confidence intervals. ^c^ The proportion of the total effect due to each component. ^d^ The sum of the controlled direct effect and reference interaction effect. ^e^ the sum of the mediated interaction effect and pure indirect effect.

## Data Availability

The data presented in this study are available on request from the corresponding author.
